# Temperature-dependent Developmental Plasticity and Its Effects on Allen’s and Bergmann’s Rules in Endotherms

**DOI:** 10.1093/icb/icad026

**Published:** 2023-05-09

**Authors:** Joshua K R Tabh, Andreas Nord

**Affiliations:** Lund University, Department of Biology, Section for Evolutionary Ecology, Sölvegatan 37, SE-223 62, Sweden; Lund University, Department of Biology, Section for Evolutionary Ecology, Sölvegatan 37, SE-223 62, Sweden

## Abstract

Ecogeographical rules, describing common trends in animal form across space and time, have provided key insights into the primary factors driving species diversity on our planet. Among the most well-known ecogeographical rules are Bergmann’s rule and Allen’s rule, with each correlating ambient temperature to the size and shape of endotherms within a species. In recent years, these two rules have attracted renewed research attention, largely with the goal of understanding how they emerge (e.g., via natural selection or phenotypic plasticity) and, thus, whether they may emerge quickly enough to aid adaptations to a warming world. Yet despite this attention, the precise proximate and ultimate drivers of Bergmann’s and Allen’s rules remain unresolved. In this conceptual paper, we articulate novel and classic hypotheses for understanding whether and how plastic responses to developmental temperatures might contributed to each rule. Next, we compare over a century of empirical literature surrounding Bergmann’s and Allen’s rules against our hypotheses to uncover likely avenues by which developmental plasticity might drive temperature-phenotype correlations. Across birds and mammals, studies strongly support developmental plasticity as a driver of Bergmann’s and Allen’s rules, particularly with regards to Allen’s rule. However, plastic contributions toward each rule appear largely non-linear and dependent upon: (1) efficiency of energy use (Bergmann’s rule) and (2) thermal advantages (Allen’s rule) at given ambient temperatures. These findings suggest that, among endotherms, rapid changes in body shape and size will continue to co-occur with our changing climate, but generalizing the direction of responses across populations is likely naive.

## Introduction

Phenotypic variation, both within and among species, is a key contributor to the beauty and resilience of life. In their theories of evolution, both Darwin and Wallace recognized this importance of variation (Wallace 1855; Darwin 1859, 1868) but lacked a formal understanding of how it might first arise. However, Darwin speculated that traits within individuals—or otherwise identical individuals—were likely malleable and varied according to environmental context (reviewed in Winther 2000). Today, this speculated process is best known as “phenotypic plasticity” and is widely understood as a primary strategy to cope with, or even exploit, novel or changing environments (see, for example, Bradshaw 1965; West-Eberhard 1989; Brooker et al. 2022).

Some of the most striking displays of phenotypic plasticity occur in response to temperature. In the Chinese primrose (*Primula sinensis*), flowers that develop red at 20°C emerge white at 30°C, regardless of parentage (Baur 1919). Similarly, five-spotted hawkmoth larvae (*Manduca quinquemaculata*) from the same brood develop black when raised at a mild ambient temperature (<20°C) but bright green when raised in the warmth (>28°C; Suzuki and Nijhout 2006). In fish, Atlantic halibut (*Hippoglossus hippoglossus*) raised in warmer waters (>10°C) accelerate growth so rapidly that they can weigh more than twice that of conspecifics held in cooler waters (6°C) by six months of age (Jonassen et al. 1999). These examples not only highlight the profound consequences of phenotypic plasticity on life, but also that the thermal environment during development can, and often does, play a direct role in mediating its occurrence.

Across natural environments, whether plastic responses to temperature can explain variations in species form has been questioned for decades but remains debated (Hansson 1985; Sebens 1987; Teplitsky et al. 2008; discussed in Yom-Tov and Geffen 2011). However, correlations between the thermal environment and both the size and shape (specifically extremity length) of endothermic animals have been known since the nineteenth century (Bergmann 1847; Allen 1877), providing provocative fodder for speculation. These correlations, now known as Bergmann’s rule (or sometimes “James’ Rule” intra-specifically; Blackburn et al. 1999), and Allen’s rule, have since been observed at both inter-specific (e.g., Ashton et al. 2000; Meiri and Daya 2003; Rodríguez et al. 2008; Symonds and Tattersall 2010; Alhajeri et al. 2020; Benítez-López et al. 2021; McQueen et al. 2022; Weeks et al. 2023) and intra-specific levels (e.g., James 1970; Ashton 2002; Freckleton et al. 2003; Benítez-López et al. 2021; McQueen et al. 2022). Although traditional explanations for both rules are generally genetic (i.e., with natural selection favoring body sizes and shapes that reduce heat loss in the cold and increase heat loss in the warmth; Mayr 1956), that each are sometimes evident within species suggests that phenotypic plasticity, in addition to fixed genetic effects, may also contribute to their occurrence. Unfortunately, the majority of studies pertaining to Bergmann’s and Allen’s rules have focused on their validity and physiological implications (see e.g., Scholander 1955; Mayr 1956; Geist 1987; Geist 1990;Meiri and Daya 2003; McNab 2010; Gutiérrez-Pinto et al. 2014), thus leaving knowledge about their mechanistic drivers comparatively less developed (but see Serrat 2007).

In this conceptual paper, we first review over a century of empirical literature testing the hypothesis that plastic responses to the thermal environment, specifically during post-natal development, give rise to intra-specific variants of Bergmann’s rule and/or Allen’s rule. While we recognize that plasticity during adulthood—or “phenotypic flexibility” (Piersma and Drent 2003)—may also contribute to the manifestation of these rules (e.g., Gosler 1987), we have chosen to focus our discussion on plastic effects during development alone owing to evidence emphasizing this life stage as a critical window for shaping the final structure and size of many vertebrates (see Wells 2014). Together, we base our discussion around both novel and traditional hypotheses describing how this plasticity might operate, and which precise phenotypes might be expected across temperatures under each. Our intent in doing so is not to exhaustively *test* how the thermal environment during development impacts body size (similar to Weeks et al. 2022) and extremity length; rather, our goal is to create a theoretical framework with which: (1) an influence of the thermal environments on endotherm size and shape—as they are understood through ecogeographical rules—might be critically evaluated, and (2) future empirical studies seeking to uncover the mechanisms driving Bergmann’s and Allen’s rules may best be oriented.

### Bergmann’s rule

Bergmann’s rule, that endotherms living in warm environments are usually smaller than their congeners in cold environments, is arguably the most well-known and hotly disputed of all ecogeographical rules. While some of this disputation surrounds the validity of the rule itself (see above), much is also semantic, and reduces to disagreements about how Bergmann’s ideas should be correctly interpreted (see Watt et al. 2010; Meiri 2011). Bergmann himself reported that a negative correlation between body size and environmental temperature (proxied by latitude) was most apparent when observed *across* species of closely related endotherms, despite first predicting a more obvious trend within species (Bergmann 1847; discussed in Watt et al. 2010). Several decades later, Rensch (1938) argued that Bergmann’s ultimate explanation—viz. that larger animals have higher capacities for heat retention—should have equal relevance within species. Although both inter- and intra-specific variants of Bergmann’s rule could be explained by selective responses to temperature, or even range shifts in animal populations over time, intra-specific variants present the possibility that temperature-body size correlations are also explained by plastic responses to the thermal environment. Given our interest in plasticity as a driver of ecogeographical rules, we have therefore chosen to focus our paper on intra-specific versions of Bergmann’s rule.

### A framework for how temperature-dependent, developmental plasticity affects body size

**Fig. 1:**
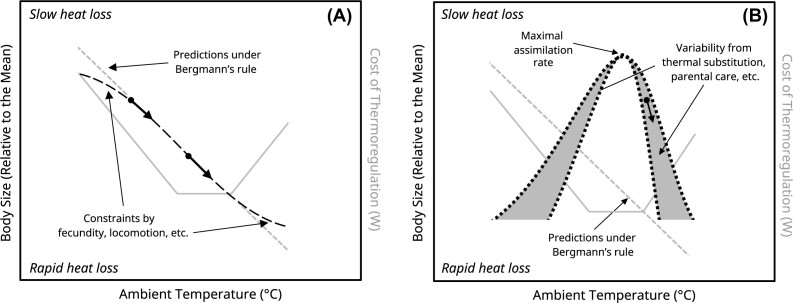
Predicted effects of ambient temperature on body size of developing endotherms under two plastic hypotheses of Bergmann’s rule. Panel **(A)** outlines predicted effects under the Thermal Advantage Hypothesis, and panel **(B)** outlines predicted effects under the Energy Efficiency Hypothesis. Black lines indicate patterns of relative body size (left *y*-axis) for a model endotherm, and gray lines indicate expected costs of thermoregulation (right *y*-axis) for the same species. Black dots represent two conspecific endotherms, and arrows leading from the dots represent predicted changes in their body size in response to a warming environment. The classic prediction of Bergmann’s rule (i.e., a negative linear correlation between ambient temperature and body size) is displayed with dashed gray lines, and expected rates of heat loss for a given relative body mass (e.g., rapid or slow) are indicated on each panel. Conformation with Bergmann’s rule is likely constrained by numerous biological processes at extreme small and large body sizes; a select set of examples (i.e., fecundity and locomotion) are provided in the figure panel. The thermoneutral zone (TNZ), where costs of thermoregulation are minimal and independent of ambient temperature, is intentionally narrow to emphasize predictions at temperatures both below and above the lower- and upper-critical temperatures, respectively (delimiting the TNZ), where most endotherms are likely to reside (see Škop et al. 2020 for an example). Note that the exact shape of curves and position of inflection points are hypothetical and will likely vary between both species and environments.

Arguably the most parsimonious route by which ambient temperature might directly influence body size, and thus give rise to Bergmann’s rule, is by shaping rates and durations of growth during post-natal development (together, “cumulative growth”). In ectotherms, such an effect—known as the “temperature-size rule”—is well supported (Walters and Hassall 2006), and its mechanistic drivers are becoming clearer (Verberk et al. 2021). Whether and how a similar effect may arise in endotherms, however, is currently unknown. In line with classic mechanisms proposed by Bergmann (1847) and Rensch (1938), increases in cumulative growth in the cold and decreases in cumulative growth in the warmth may reflect selection on the efficiency of heat exchange at given temperatures (henceforth, the “Thermal Advantage Hypothesis;” Box 1). A likely alternative, however, is that changes in cumulative growth across ambient temperature occur to increase efficiency of energy use during post-natal development (henceforth, the “Energy Efficiency Hypothesis;” Box 1; see Parsons 2005 for the fitness value of energy efficiency). This distinction between mechanisms is critical, since precisely how body size should vary across ambient temperatures is likely to differ under each. Under the Thermal Advantage Hypothesis, cumulative growth and ultimately relative body size should correlate linearly and negatively with ambient temperature, regardless of concurrent thermogenic or thermolytic costs, until constraints imposed by other fitness-related traits emerge (e.g., fecundity and locomotion; Alisauskas and Ankney 1990; Shaeffer and Lindstedt 2013; see Boyer et al. 2010; Fig. 1A). Under the Energy Efficiency Hypothesis, however, correlates between cumulative growth (or relative body size) and ambient temperature should instead represent a right-skewed quadratic with maximum values (i.e., the apex) occurring at, or near, the temperature at which maximum energy assimilation rate is achieved (Fig. 1B). The temperatures at which net growth or relative size becomes negative (i.e., *x*-intercepts) should then lay where either the energetic costs of thermoregulation begin to compete with, and compromise, those of growth, or where growth is stunted by heat-induced cellular damage (Fig. 1; see Ørstedt et al. 2022).

Box 1 **How may plastic responses to developmental temperatures explain Bergmann’s rule in endotherms?**

Intra-specifically, Bergmann’s rule states that the body size (and thus, surface-area-to-volume ratios) of conspecific endotherms is typically larger in cooler environments than in warmer environments. This negative correlation between size and ambient temperature is generally thought to reduce the costs of thermoregulation by slowing rates of heat loss in the cold, and increasing rates of heat loss in the warmth.

We contrast two hypotheses explaining how plastic responses to temperature during post-natal development may lead to Bergmann’s rule-like patterns within endothermic species: the Thermal Advantage Hypothesis, and the Energy Efficiency Hypothesis. The Thermal Advantage Hypothesis posits that cooler temperatures lead to increases in cumulative growth during development, thus increasing adult body size and decreasing the total costs of thermoregulation at maturity. Here, increases in growth in the cold (and, therefore, final body size) occur despite, and concurrent with, higher energetic costs of heat production (Fig. 1A). A seldom-discussed nuance to this hypothesis, and Bergmann’s rule itself, is that correlations between body size and ambient temperature should diminish at extreme temperatures, when constraints from other fitness-related traits (e.g., fecundity and locomotion) are imposed on body size (Fig. 1A). Contrasting the Thermal Advantage Hypothesis, the Energy Efficiency Hypothesis posits that ambient temperature influences cumulative growth during development by: (1) establishing the amount of resources available for growth by first setting energy cost of thermoregulation, and (2) determining the rate at which acquired energy can be assimilated. Under this hypothesis, the relationship between body size (via cumulative growth) and ambient temperature is best represented by a skewed quadratic, with apex at the temperature of maximal energy assimilation and *x*-intercepts near the upper and lower inflection points of a species’ prescriptive or thermoneutral zone (Mitchel et al. 2018; Fig. 1B). The term “near” is emphasized to acknowledge that other physiological parameters, including heat substitution from growth, parental care strategies, and strategies for mass deposition (i.e., muscle vs. fat; see Heath 1983) are likely to influence their true locations. In Fig. 1B, this uncertainty is indicated by light-gray bands. Skewness of the temperature-growth relationship is negative, with decreases in growth occurring faster at high ambient temperatures, since endotherms are often heterothermic or poikilothermic during development (see Whittow and Tazawa 1991; Geiser 2008), and rates of metabolic processes increase most rapidly with increasing tissue/body temperatures (see Mundim et al. 2020).

If Bergmann’s rule is explained by plastic responses to ambient temperature, and such plastic responses occur to confer thermal advantages, one may predict that increases in ambient temperature during development should cause unanimous decreases in body size and vice versa, until constraints on size are imposed by other fitness-related traits (discussed above; Fig. 1A). However, if plastic responses occur to increase the efficiency of energy use, a more complex pattern in response to warming temperatures should emerge. More specifically, if ambient temperatures are usually high during development (relative to a range of developmental temperatures that are the most conducive for growth), then further increases in temperature should impose a decrease in body size. By contrast, if ambient temperatures are usually low during development (again, relative to the optimal range of developmental temperatures), increases in temperature should instead impose an increase in body size (Fig. 1B). In the context of a warming climate, these two hypotheses present very different responses with respect to individual phenotype.

Below, we interpret empirical literature within this theoretical framework and question whether there is: (1) evidence of a plastic origin to Bergmann’s rule in endotherms at all, and (2) indication that any plastic origin to Bergmann’s rule conforms more closely with the Thermal Advantage Hypothesis or the Energy Efficiency Hypothesis. To facilitate these ends, findings are discussed qualitatively and with a focus on effect sizes (in % change). Thermal effects imposing less than an arbitrary 5% change in body size are considered weak.

### Evidence for direct effects of developmental temperature on body size across endotherms

Controlled experiments, whereby ambient temperature alone is varied during development, remain the gold standard for evaluating a plastic origin to ecogeographical rules. In one of the first of its kind, Sumner (1909) reared newly-weaned, captive-born mice (*Mus musculus*) in either cold environments (approximately 6°C) or warm environments (approximately 26°C) while monitoring body mass throughout development. In contrast to Bergmann's rule, the average mass of cold- and warm-reared mice was strikingly similar at 6 weeks of age (i.e., at the end of maximum growth velocity; Kurnianto et al. 1997), with cold-reared mice being less than 0.5 g (2%), heavier than their warm-reared conspecifics. Many years later, Ashoub (1958) corroborated these findings, showing that, albeit subjectively, wild-origin mouse pups reared at 10°C appeared to develop “normally.” Among more modern research, similarly limited effects of cold exposure on body size development have been well supported. Ballinger and Nachman (2022), for example, found that the average mass of wild-derived house mice reared at 5°C was within 0.3 g (again, 2%) of those reared at 21°C, even when compared between full siblings of the same sex. Lower ambient temperatures (-3°C) did little to change this effect, with the masses of adult, cold-reared mice being only 4% higher than warm-reared mice (23°C; Barnett and Dickson 1984). Even more surprisingly, Serrat et al. (2008) reported a *decrease* in body mass (6%) among cold-reared mice (7°C) relative to warm-reared mice (27°C) at 12 weeks of age. Such findings (specifically, those of Serrat et al. 2008) are not only inconsistent with expectations of Bergmann’s rule, but directly oppose them. Beyond mice, still other mammalian studies have repeatedly shown negligible to weak effects of developmental cold exposure on mature body mass, suggesting that enhanced growth in these conditions—vis-à-vis classic interpretations of Bergmann’s rule—is hardly universal (pigs, *Sus scrofa*: Weaver and Ingram 1969; domestic rats, *Rattus norvegicus*, Quinn 1978; Albustanji et al. 2019; fat-tailed dunnarts, *Sminthopsis crassicaudata*, Riek and Geiser 2012; yellow-footed antechinuses, *Antechinus flavipes*, Stawski and Geiser 2020; see Heath 1984 for an in-depth review of early literature).

In birds, a similar picture of how developmental cold exposure relative to thermoneutral conditions influences adult phenotype is emerging. In Japanese quail (*Coturnix japonica*), Burness et al. (2013) reported a minimal (ca. 1%) effect of rearing young at 15°C throughout early development (5–51 days of age) on body mass at maturity (56–84 days) when compared with 30°C controls, despite subtle differences in mass earlier on. Likewise, exposure to post-natal cooling bouts (20°C relative to 30°C) led to no detectable changes in the adult mass of domestic chickens (*Gallus gallus;*Mujahid and Furuse 2009; but see May and Lot 2001). Further lowering ambient temperatures in cold exposure treatments, however, appears to elicit slightly different results in both species. In Japanese quail, for example, we recently observed that rearing young at 10°C from hatching onward leads to negative effects on adult body mass, not positive, with cold-reared birds weighing 7% less at maturity than those reared in the warmth (30°C; Persson E., Tabh J. K. R., Nord A., et al., unpublished data). Snedecor (1971) reported a similar end with the body mass of domestic chickens being 10% higher when reared at intermediate (25°C) rather than cool (15°C) temperatures. Such negative effects of developmental cold exposure have also been supported in at least two other avian species (Muscovy ducks, *Cairina moschata*, and great tits, *Parus major;*Teulier et al. 2014; Rodríguez and Barba 2016a; but see negligible effects of cycling cold temperature on body size in chickens; Swain and Farrell 1975).

Contrasting results from cold-exposure studies, those obtained from experimental heat exposures (again, relative to thermoneutral conditions) generally do support expectations of Bergmann’s rule. In mice, for example, young raised at 35°C after weaning were 11% lighter than those reared at 25°C in otherwise similar environments (Sundstroem 1922a, 1922b). Similarly, guinea pigs (*Cavia porcellus*) raised at 36°C were 9% smaller at one week of age than those raised at 21°C (Adamsons et al. 1969), and domestic pigs exposed to cycling heat stressors within their second week (between 32 and 38°C) were 0.4 kg (8%) lighter at weaning than controls (25.4°C; Johnson et al. 2018). In birds, a recent review of literature published over the last half-century reported that 9 of 15 relevant studies revealed a negative effects of heat exposure during development on the body size of young at fledging or maturity (Weeks et al. 2022). While intriguing, the varied nature of metrics used to measure “body size” (e.g., tarsus length, wing length, body mass) may limit the study’s interpretability in the context of Bergmann’s rule, particularly since some metrics may have greater relevance to Allen’s rule (e.g., tarsus length; discussed below). Regardless, experimental studies monitoring the body mass of birds throughout post-hatch development often show a negative effect of heating on growth or final mass (e.g., May and Lot 2001; Rodríguez and Barba 2016a, albeit non-significant; Marchini et al. 2011; Andreasson et al. 2018; but see Ernst et al. 1984). As with cold-exposure studies, however, this negative effect is not always evident and is, in some cases, reversed (see, for example, Herrington and Nelbach 1942; Dawson et al. 2005; Pérez et al. 2008; Ton et al. 2021), even among observational studies (Teplitsky et al. 2008; Shipley et al. 2022). Nevertheless, such directional inconsistencies appear less common among experimental warming studies than experimental cooling studies.

### Bergmann’s rule in light of developmental plasticity literature

Although the precise timing of heat- or cold-exposures during development, as well as resource abundance, may generate some noise in the findings discussed above (see Knudsen 1962; Serrat 2013; Nord and Giroud 2020), evidence across both birds and mammals generally support an effect of post-natal heat exposure, but less so cold exposure, on final body size. Still, when viewed across a sufficiently broad range of ambient temperatures, it is nonetheless likely that thermal sensitivity of body size during development does contribute to Bergmann’s rule-like patterns. Perhaps more interestingly, however, the varying and non-linear responses of endotherms to experimentally cooled or heated environments highlight that plastic contributions to Bergmann’s rule are unlikely to be explained by selection for thermal benefit alone (i.e., the Thermal Advantage Hypothesis). Instead, these findings better align with the hypothesis that plastic contributions to Bergmann’s rule are driven by selection to increase efficiency of energy use in a given thermal environment (i.e., the Energy Efficiency Hypothesis; Fig.   1B). Indeed, under this hypothesis, body size responses to a temperature challenge should not be linear and should depend on the degree to which the challenge shifts development within, or outside temperature zones that are prescriptive for growth (*sensu*Mitchell et al. 2018; Fig. 1B). Supporting this prediction, the body mass of tree swallow nestlings (*Tachycineta bicolor*) increased when experimental heating raised developmental temperatures to within thermoneutrality (i.e., 30°C; Williams 1988). Moreover, pushing developmental temperatures into ranges that likely increased costs of heat dissipation and decreased energy assimilation rates led to *decreases* in body mass of other avian species (observed in Andreasson et al. 2018 and Johnson et al. 2018, where experimental heating raised developmental temperatures well above thermoneutrality for their study species; O'Connor 1975; Huynh 2005). With these findings in mind, we speculate that temperature-mediated plasticity should not induce unanimous decreases in body size when temperatures rise (e.g., Fig. 1A), as is often predicted for endotherms in a climate warming scenario (e.g., Sheridan and Bickford 2011; Youngflesh et al. 2022). Rather, plastic responses to a warming world may well manifest in a more complex and nuanced manner, with high-latitude or otherwise cold-exposed populations increasing in cumulative growth and body size (consistent with Meiri et al. 2009 and Boutin and Lane 2014), and already heat-exposed populations decreasing. Of course, we recognize that other selective processes (e.g., relaxed selection on body size in warm winters) probably do influence how body size might respond to warming or changing climates (Ozgul et al. 2009; Ballinger and Nachman 2022; but see Teplitsky et al. 2008). However, widespread support for plastic responses to developmental temperature indicate that such should not be ignored when seeking to understand the emergence of Bergmann’s rule and species-level responses to climatic change.

### Allen’s rule

Allen’s rule states that endotherms living in colder environments tend to have shorter bodily extremities than those living in warmer environments. Unlike Bergmann’s rule, the intra- or inter-species specificity of this particular rule has been subject to relatively little debate. Although Allen restricted his observations to phenotypic trends within species, his original writings did not exclude the possibility or similar trends emerging across species of a phylogenetic grouping (see Allen 1877). This possibility has now been supported with several broad-scale studies on birds and mammals (e.g., Nudds and Oswald 2007; Symonds and Tattersall 2010; Alroy 2019; Alhajeri et al. 2020; but see Gohli and Voje 2016). Functionally, Allen’s rule is understood as a mechanism to reduce the loss of costly body heat in the cold and increase the loss of damaging body heat in the warmth (i.e., by reducing or increasing relative body surface area, respectively). However, whether this function is achieved through natural selection on, or plasticity of, extremity length is unclear (see Mayr 1956; Gohli and Voje 2016).

### Models for how developmental temperature and plasticity affect extremity length in endotherms

Allen himself speculated that variations in extremity length within species were caused by plastic responses to their local environments—a quite different view from that held by Bergmann. Indeed, in the introduction of his seminal work, Allen (1877; p. 1–2) states: “*. . .[my conclusions] show that other influences than natural selection operate powerfully in the differentiation of specific forms, and that geographical causes share more largely in the work than naturalists have heretofore been prepared to admit*.” Although no empirical evidence is provided to support his hypothesis, the observation that the pelage of domestic sheep thickens in response to cooler climates is offered as allegorical rationale. This deduction is notable since it reveals that selection favoring plastic responses to temperature, or adaptive phenotypic plasticity, is arguably best aligned with Allen’s conclusions. More specifically, plasticity to reduce extremity length in the cold and increase extremity length in the warmth may be selected to decrease and increase heat loss in each environment, respectively (i.e., the “Thermal Advantage Hypothesis” under Allen’s rule; Box 2). Yet, an obvious alternative hypothesis is that any plastic changes in extremity length induced by the thermal environment are merely byproducts of other adaptive or non-adaptive responses to temperature. Under this hypothesis (henceforth, the “Exaptation Hypothesis”; Box 2), temperature-mediated plasticity of extremity length is not a result of natural selection for thermal advantages *per se*, but nevertheless still provides energetic benefits within some thermal environments (similar to an evolutionary spandrel; Gould and Lewontin 1979).

Box 2 **How may temperature-dependent, developmental plasticity explain Allen’s rule in endotherms?**

Allen’s rule states that the bodily extremities of both conspecific and heterospecific endotherms are usually shorter in cooler environments than in warmer environments (Allen 1877). Like Bergmann’s rule, Allen’s rule is typically explained in thermoregulatory terms, with shortened extremities enhancing heat retention in the cold and elongated extremities enhancing heat loss in the warmth.

**Fig. 2:**
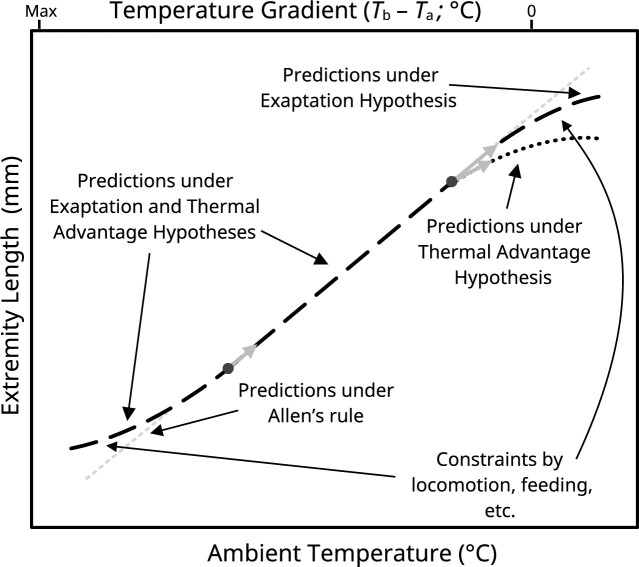
Predicted effects of ambient temperature on the developmental elongation of bodily extremities in endotherms. The dotted gray line indicates the classic expectation under Allen’s rule, that there is a positive linear correlation between ambient temperature and extremity length. Similar to Bergmann’s rule, complete conformation with Allen’s rule is likely to be constrained by certain biological processes at size extremes; two relevant examples (i.e., locomotion and feeding) are provided. Black dots represent two model endotherms, and gray arrows leading from dots represent predicted changes in their extremity lengths in a warming environment. These dots highlight different growth trajectories depending on whether extremity elongation follows predictions under the Thermal Advantage Hypothesis or the Exaptation Hypothesis. Distinctions between these trajectories are predicted to manifest at high ambient temperatures alone, when dry heat loss to the environment becomes less significant and ultimately reversed (i.e., when the environment is warmer than the animal). For example, if extremities lengthen in the warmth under the Thermal Advantage Hypothesis, then there should be no further increase in length when ambient temperature (*T*_a_) surpasses body temperature (*T*_b_) and dry heat loss becomes negative (indicated by 0 on the secondary *x*-axis).

Most parsimoniously, increasing ambient temperatures may lead to plastic elongation of extremities throughout development via either: (1) adaptive plasticity to reduce heat loss in the cold and increase heat loss in the warmth (the Thermal Advantage Hypothesis), or (2) direct, positive, and not-always-adaptive effects of temperature on cell proliferation and metabolism (i.e., via q10 effects; the Exaptation Hypothesis). Under the Thermal Advantage Hypothesis, elongation of extremities is expected to slow, stop, or even reverse when benefits to heat dissipation are no longer evident (indicated by a zero body-to-ambient temperature gradient on the secondary *x*-axis in Fig. 2). By contrast, under the Exaptation Hypothesis, extremities should elongate with increasing ambient temperatures regardless of any heat dissipation benefits. In the cold, both hypotheses predict a continuous decrease in extremity length since such decreases may occur either as a direct effect of dry heat loss or an indirect effect of selection to decrease extremity surface area and, thus, heat loss. Phenotypic trends at these temperatures, therefore, are not informative when seeking to distinguish between each hypothesis. Similar to body size, extremity lengths in temperature extremes are likely to be constrained by other fitness-related traits such as locomotion and feeding.

There are likely several routes by which temperature might influence extremity length indirectly, or as a byproduct. One of the simplest and best described is through q10 effects on cellular metabolic process and cell proliferation. For example, the rate of chondrocyte division (and hence bone elongation) has been shown to increase with increasing tissue temperatures, even *ex vivo* when housing temperatures are fixed above expected body temperatures (Serrat et al. 2008). While some of this correlation may still be explained by adaptive adjustments in, for example, the cellular machinery responsible for nutrient uptake and delivery, mere q10 effects are nonetheless also probable (reviewed in Serrat 2014). Such effects would have important implications for phenotypic responses at high ambient temperatures where extremity elongation could become exacerbated beyond that advantageous for dry heat loss (i.e., further lengthening even once ambient temperature exceeds body temperature), unless the lengthening response is constrained by accumulation of cellular damage in the heat (again, see Ørsted et al. 2022). In this way, the Thermal Advantage and Exaptation Hypotheses should yield different predictions regarding the effect of ambient temperature on extremity length. Specifically, under the Thermal Advantage Hypothesis, elongation of extremities in the warmth should only occur insofar as advantages to dry heat loss are provided (i.e., when ambient temperature is below body temperature and heat can be lost non-evaporatively) and should diminish thereafter (Fig. 2). By contrast, under the Exaptation Hypothesis, where q10 effects are likely contributors to extremity growth, elongation of extremities in the heat should continue as temperatures rise regardless of whether advantages to heat loss exist or not (Fig. 2) and will be truncated only when selection against extremity length for non-thermoregulatory reasons appear. In response to cold, predictions under both hypotheses are similar since stunting of extremity growth should continue to provide thermal advantages even at extreme low temperatures (Fig. 2). Although responses to temperature under each hypothesis are likely to be bound by functional constraints (e.g., locomotion or feeding), differences in their expected consequences nonetheless paint unique pictures of how endotherms may change in a warming world.

Below, we review empirical literature seeking to uncover a role of developmental plasticity in dictating Allen’s rule within species and discuss these findings in light of the Thermal Advantage and Exaptation Hypotheses to the ontogeny of temperature-extremity length relationships across endotherms.

### Evidence for plastic effects of developmental temperature on extremity lengths

In mammals, exposure to cold during development often elicits dramatic effects on the growth of the tail, limbs, and other bodily appendages (e.g., ears and antlers). In one of the most remarkable examples of this, Thorington (1970) observed a 32% decrease in the tail lengths of white-footed mice (*Peromyscus leucopus*) reared at 16°C relative to 27.5°C by 12 weeks of age, independent of cold-induced changes in body size. Similar cold-induced reductions in tail growth have also been observed in studies of domestic mice. Knudsen (1962), for example, reported a 30% reduction in tail length among eight-week-old mice reared at 18°C relative to 32°C. Moreover, Sumner (1909), Barnett (1965), and Barnett and Dickson (1984) each observed reductions in tail length exceeding 5% among mature mice that were reared below 10°C relative to near-room temperatures (23–25°C). In one of these cases (Barnett 1965), the stunting effects of cold exposure correlated with a decrease in both the absolute number of caudal vertebrae and their individual length. Thus, temperature effects on extremity growth may extend beyond modifications to cartilaginous or muscular tissues (confirmed by Al-Hilli and Wright 1983; see Serrat et al. 2014 for an in-depth review of this topic). At the level of the limbs and ears, cold-induced growth restrictions are equally well supported. Lowering ambient temperatures to 5°C after weaning elicited a 10% reduction in femur length and a 25% reduction in ear surface area of domestic pigs at 88 days of age when compared with warm-raised controls (35°C; Weaver and Ingram 1969). In rats, raising young from weaning at 3–5°C relative to 18–28°C also led to 5% reductions in tibial length, 7% reductions in third metatarsal length, and other notable but unquantified declines in radial, ulnar, and ear length at maturity (Lee et al. 1969; Riesenfeld 1973; see Villarreal et al. 2007 for similar findings). Further findings in domestic mice are also comparable (Serrat et al. 2008). These lesser reductions in limb length, relative to those reported for tails and ears, are noteworthy, but can probably be explained by an earlier emergence of functional constraints when key constituents of the locomotory apparatus are modified, leaving fewer possibilities for developmentally plastic changes in some appendages compared to others.

In birds, empirical studies evaluating a role of ambient temperature on extremity growth are comparatively few. In great tits (*P. major*), cooling of nests by 5°C after hatching led to a weak 4% reduction in tarsus length at 15 days of age (Rodríguez and Barba 2016a), and in Japanese quail, rearing at 7°C relative to 24°C led to a 2.5% reduction in tarsus length by maturity (Krijgsveld et al. 2003). Although the bill is recognized as a potentially important structure for avian thermoregulation (Tattersall et al. 2017) and known to follow Allen’s rule (Symonds and Tattersall et al. 2010; Fan et al. 2019; Romano et al. 2020), we are only aware of two studies using experimental methods to test an effect of rearing temperatures on adult bill length (NeSmith 1985, as discussed in James 1991; Burness et al. 2013). In one study, cold temperatures during development reportedly caused a qualitative reduction in bill length near fledging (in Red-winged blackbirds, *Agelaius phoeniceus*; NeSmith 1985), while in the other, rearing temperature elicited a negative but weak effect (∼3% reduction) on bill length at maturity (in Japanese quail; Burness et al. 2013). Most observations among mammals, but less so birds, therefore appear to indicate a negative effect of low developmental temperatures on the elongation of extremities, which could contribute to morphometric clines recognized as Allen’s rule.

Studies measuring how extremity lengths respond to heat exposure in both birds and mammals are also scarce, particularly with heat treatments nearing or exceeding body temperature. However, in one early study (Przibram 1925), 11-week-old rats that had been reared in ambient temperatures between 5°C and 40°C showed an almost linear increase in relative tail length with increasing temperature, even when ambient temperatures exceeded body temperatures typical for this species (i.e., 37–39°C; Poole and Stephenson 1977). In another study, unilateral surface heating at 40°C throughout development led to significant increases in limb and ear length of 5-week-old mice when compared with mice unilaterally heat-treated at 30°C (Serrat et al. 2015). These findings suggest that plastic contributions to extremity length hold even at ambient temperatures above body temperature (i.e., as predicted by the Exaptation Hypothesis; Fig. 2). In stark contrast, however, three studies in birds reported no effect of experimental heating in the nest on tarsus length near fledging (Dawson et al. 2005; Rodríguez and Barba 2016b; Andreasson et al. 2018). Moreover, although domestic chickens raised at 35°C displayed longer legs than those raised at 15°C, leg lengths were still comparable to those raised at 25°C by 5 weeks of age, indicating that cold stunts, but heat does not affect, extremity length in this species (Snedecor 1971). These studies hint that plastic changes in extremity length following heat exposure might be reduced, or even negated above a certain threshold temperature (predicted by the Thermal Advantage Hypothesis to Allen’s rule, Fig. 2). Although possibly confounded by parental behavior, that several field observations have supported such a conclusion (e.g., a lack of extremity elongation in extreme heat) is intriguing (see Cunningham et al. 2013; Pipoly et al. 2013; Andrew et al. 2017).

### Allen’s rule in light of developmental plasticity literature

Findings from experimental literature strongly support an effect of ambient temperature during post-natal development on the elongation, or shortening, of bodily extremities in birds and mammals. This effect, in most cases, is consistent with intra-specific correlations between ambient temperature and extremity length known as “Allen’s rule,” particularly in response to low and moderate developmental temperatures (in keeping with both the Thermal Advantage and Exaptation Hypotheses). However, although empirical studies are limited, available evidence appears to indicate a reduced, and even negated effect of high heat load on the lengthening of extremities during development. These observations are not consistent with the Exaptation Hypothesis, but do support the Thermal Advantage Hypothesis (Fig. 2). Such findings are important since they suggest that: (1) plastic changes in extremity length consistent with Allen’s rule may well be adaptive responses to reduce heat loss in the cold and increase heat loss in the warmth, and (2) species developing in hot environments may already display maximal extremity lengths for their body sizes and change little in response to further warming.

Similar to body size, extremity lengths may in part be explained by inheritance of, and selection on, fixed phenotypes (e.g., Cheung and Parker 1974; Alatalo and Lundberg 1986). Nevertheless, in view of substantial plasticity in extremity length when offspring thermal environments are shifted (discussed above), the contributions of such to Allen’s rule need not be in isolation; indeed, studies in mice have concluded the same (e.g., Serrat 2007; Ballinger and Nachman 2022). For many endothermic species, this lack of fixity in extremity lengths implies that changes in response to a warming climate may well be rapid (consistent with Ryding et al. 2021). Critically, however, evidence also suggests that the extent to which these changes occur will probably depend upon the range at which ambient temperature is raised in a species’ breeding environment, and whether extremity lengths are already maximized for a given species.

## Outlook

Numerous empirical studies across endotherms indicate that the development of both body size and extremity length is labile and can differ according to the post-natal thermal environment. Broadly speakings, this lability often recapitulates the classic phenotypic clines known as Bergmann’s and Allen’s rules. However, a wide body of evidence also suggests that the precise shapes of temperature-dependant changes in body size and form are not linear. Instead, phenotypic responses to developmental temperature are probably more nuanced and dependent on both environmental context (i.e., range-specific ambient temperatures) and individual-level factors (i.e., intrinsic temperature tolerance and energy available for growth). Accordingly, while increases in ambient temperature often do cause plastic reductions in body size and increases in extremity length (following Bergmann’s and Allen’s rules, respectively), these changes may be lost or even reversed at relatively low and extremely high ambient temperatures, respectively. With this in mind, we argue that although warming climates may well lead to rapid changes in the morphology of endotherms, consistent and generalized responses of shrinking body sizes and elongating extremities are doubtful.

Although supported by decades of empirical literature, we acknowledge that our mechanistic hypotheses and conclusions remain largely theoretical. Thus, to better interrogate each, we suggest that future research be directed toward three main areas:

1. *The functional or adaptive significance of Bergmann’s and Allen’s rule*. Changes in body size and extremity length that recapitulate ecogeographical rules and track a warming climate are expected to: (i) endow individuals with thermal/energetic benefits; and (ii) carry implicit fitness advantages (e.g., Youngflesh et al. 2022). However, both the thermal and fitness advantages of conforming with Bergmann’s and Allen’s rules remain surprisingly understudied and may even be insignificant when compared from those obtained by adjusting thermoregulatory behaviors, blood flow patterns, heat production, and evaporative cooling (Scholander 1955; McNab 1971, 2010; Briscoe et al. 2015; but see Steudel 1994). For this reason, we recommend that future studies critically evaluate the thermoregulatory benefits of each rule and attempt to put their findings into a context of survival and reproductive performance in a changing world. Such studies could be achieved either in the laboratory using respirometry, or in the wild by combining infrared thermography and biophysical modeling (McCafferty et al. 2011). Addressing whether an influence of developmental temperature on body size and shape can be generalized across sexes would also be valuable, particularly since patterns of selection on these traits may differ per sex for non-thermoregulatory reasons (e.g., fecundity- or propagule size-selection in females; cf. Ronget et al. 2018).
2. *The relative contributions of environmental, genetic, and genotype-by-environment effects toward Bergmann’s and Allen’s rule*. Our review highlights that environmental effects contribute to temperature-to-phenotype relationships known as Bergmann’s and Allen’s rules. Still, genetic contributions toward each are also well supported (Teplitsky et al. 2008; Ozgul et al. 2009; Ballinger and Nachman 2022), and some studies have even elucidated a combination of genetic and the environmental contributions (i.e., genotype-by-environment effects; Harrison et al. 1959; Barnett 1965). To help disentangling the precise contributions of each of these effects—and thus understand how matching of body size and shape to a given thermal environment might evolve—more studies leveraging full-sib breeding designs (e.g., Ballinger and Nachmann 2022), particularly in wild taxa, are needed. Such studies could either be undertaken on wild-caught subjects reared in controlled and varying environments (see Ballinger and Nachman 2022), or could be based on reciprocal transplant experiments (possible over wide latitudinal ranges in birds; Broggi et al. 2005). Captive models can still be highly useful in this endeavor, however, only on the premise of retained thermal plasticity of phenotypic traits (Morgan et al. 2022).
3. *The proximate underpinnings of plastic responses to developmental temperature*. Our study sought to overview: (i) evidence backing plastic contributions toward Bergmann’s and Allen’s rules; and (ii) support for key hypotheses describing how, at the whole animal level, such plastic contributions might emerge. Thus, the precise physiological or molecular drivers behind any temperature-dependent plasticity that might recapitulate Bergmann’s and Allen’s rule fell beyond the scope of our study. Uncovering these drivers is a critical next step if we wish to fully appraise the whole animal-level hypotheses raised here. With respect to Bergmann’s rule, Weeks et al. (2022) recently speculated that the temperature dependence of insulin-like growth factors (namely IGF1) may influence the emergence of temperature-size relationships within avian species. Evaluating how IGFs respond quantitatively and qualitatively to ambient temperature would provide valuable insight on the likelihood of our Thermal Advantage or Energy Efficiency Hypotheses toward Bergmann’s rule (Fig. 1). With respect to Allen’s rule, *in vitro* experiments by Serrat et al. (2008) have shown that heat exposure may directly increase the elongation of extremities by speeding rates of endochondral ossification (discussed above). Assessing the strength of this effect *in vivo*, and whether it breaks down or plateaus at ambient temperatures where dry heat loss is no longer useful for thermoregulation would strongly enable comparative evaluations of the Exaptation and Thermal Advantage Hypotheses. Such assessments would require experimental approaches that separate the direct, emergent effects of temperature on tissue temperature and cell growth rate from any indirect effects of temperature perception by the animal.

Evaluating the functional significance, genetic contributions, and fine-scale mechanistic drivers of Bergmann’s and Allen’s rules are key to understanding how quickly these rules might emerge and whether they may do so adaptively. More importantly, doing so will be essential if we wish to generate accurate forecasting models for animal morphology in a warming world. We hope that the points raised in this commentary, and the practical framework concluding it will be inspiring for future research into animals, temperature, and morphological change within and beyond global warming contexts.

## Data Availability

No data were used in the construction of this manuscript.

## Notes

From the symposium “Biology at birth: the role of infancy in providing the foundation for lifetime success” presented at the annual meeting of the Society for Integrative and Comparative Biology virtual annual meeting, January 16–March 31, 2023.

## References

[bib2] Adamsons K , Blumberg E, Joelsson I. 1969. The effect of ambient temperature upon post-natal changes in oxygen consumption of the guinea-pig. J Physiol. 202:261–9. 5784287 10.1113/jphysiol.1969.sp008809 PMC1351480

[bib6] Al-Hilli F , Wright EA. 1983. The effects of changes in the environmental temperature on the growth of tail bones in the mouse. Br J Exp Pathol. 64:34–42. 6838762 PMC2040777

[bib3] Alatalo RV , Lundberg A. 1986. Heritability and selection on tarsus length in the pied flycatcher (*Ficedula hypoleuca*). Evol. 40:574–83. 10.1111/j.1558-5646.1986.tb00508.x 28556344

[bib4] Albustanji L , Perez GS, Alharethi E, Aldiss P, Bloor I, Barreto-Medeiros JM, Budge H, Symonds ME, Dellschaft N. 2019. Housing temperature modulates the impact of diet-induced rise in fat mass on adipose tissue before and during pregnancy in rats. Front Physiol. 10:209. 30894820 10.3389/fphys.2019.00209 PMC6414463

[bib5] Alhajeri BH , Fourcade Y, Upham NS, Alhaddad H. 2020. A global test of Allen’s rule in rodents. Global Ecol Biogeogr. 29:2248–60.

[bib7] Alisauskas RT , Ankney CD. 1990. Body size and fecundity in lesser Snow Geese. The Auk. 107:440–3.

[bib8] Allen JA . 1877. The influence of physical conditions in the genesis of species. Radic Rev. 1:108–40.

[bib9] Alroy J . 2019. Small mammals have big tails in the tropics. Glob Ecol Biogeogr. 28:1042–50.

[bib10] Andreasson F , Nord A, Nilsson JÅ. 2018. Experimentally increased nest temperature affects body temperature, growth and apparent survival in blue tit nestlings. J Avian Biol. 49:jav–01620.

[bib11] Andrew SC , Hurley LL, Mariette MM, Griffith SC. 2017. Higher temperatures during development reduce body size in the zebra finch in the laboratory and in the wild. J Evol Biol. 30:2156–64. 28976621 10.1111/jeb.13181

[bib12] Ashoub MER . 1958. Effect of two extreme temperatures on growth and tail-length of mice. Nature. 181:284. 10.1038/181284a0 13504163

[bib14] Ashton KG , Tracy MC, Queiroz AD. 2000. Is Bergmann’s rule valid for mammals? Am Nat. 156:390–415. 29592141 10.1086/303400

[bib13] Ashton KG . 2002. Patterns of within-species body size variation of birds: strong evidence for Bergmann’s rule. Glob Ecol Biogeogr. 11:505–23.

[bib134_1684429378728] Ballinger MA , Nachman MW. 2022. The contribution of genetic and environmental effects to Bergmann’s rule and Allen’s rule in house mice. The American Naturalist. 199:691–704. 10.1086/719028 35472023

[bib16] Barnett SA , Dickson RG. 1984. Changes among wild house mice (*Mus musculus*) bred for ten generations in a cold environment, and their evolutionary implications. J Zool. 203:163–80.

[bib15] Barnett SA . 1965. Genotype and environment in tail length in mice. Q J Exp Physiol Cogn Med Sci. 50:417–29. 5177006 10.1113/expphysiol.1965.sp001807

[bib17] Baur E . 1919. Einführung in die Experimentelle Vererbungslehre. Berlin: Gebrüder Borntraeger.

[bib18] Benítez-López A , Santini L, Gallego-Zamorano J, Milá B, Walkden P, Huijbregts MAJ, Tobias JA. 2021. The island rule explains consistent patterns of body size evolution in terrestrial vertebrates. Nat Ecol Evol. 5:768–86. 33859376 10.1038/s41559-021-01426-y

[bib19] Bergmann C . 1847. Über die verhältnisse der wärmeökonomie der Thiere zu ihrer Grösse. Göttinger Studien. 3:595–708.

[bib20] Blackburn TM , Gaston KJ, Loder N. 1999. Geographic gradients in body size: a clarification of Bergmann’s rule. Divers Distrib. 5:165–74.

[bib22] Boutin S , Lane JE. 2014. Climate change and mammals: evolutionary versus plastic responses. Evol Appl. 7: 29–41. 24454546 10.1111/eva.12121 PMC3894896

[bib23] Boyer AG , Cartron JLE, Brown JH. 2010. Interspecific pairwise relationships among body size, clutch size and latitude: deconstructing a macroecological triangle in birds. J Biogeogr. 37:47–56.

[bib24] Bradshaw AD . 1965. Evolutionary significance of phenotypic plasticity in plants. Adv Genet. 13:115–55.

[bib25] Briscoe NJ , Krockenberger A, Handasyde KA, Kearney MR. 2015. Bergmann meets Scholander: geographical variation in body size and insulation in the koala is related to climate. J Biogeogr. 42:791–802.

[bib26] Broggi J , Hohtola E, Orell M, Nilsson JÅ. 2005. Local adaptation to winter conditions in a passerine spreading north: a common-garden approach. Evol. 59:1600–3. 16153046

[bib27] Brooker R , Brown LK, George TS, Pakeman RJ, Palmer S, Ramsay L, Schöb C, Schurch N, Wilkinson MJ. 2022. Active and adaptive plasticity in a changing climate. Trends in Plant Science. 27:717–28. 35282996 10.1016/j.tplants.2022.02.004

[bib28] Burness G , Huard JR, Malcolm E, Tattersall GJ. 2013. Post-hatch heat warms adult beaks: irreversible physiological plasticity in Japanese quail. Proc Biol Sci. 280:20131436. 23884093 10.1098/rspb.2013.1436 PMC3735260

[bib29] Cheung TK , Parker RJ. 1974. Effect of selection on heritability and genetic correlation of two quantitative traits in mice. Can J Genet Cytol. 16:599–609. 4447906 10.1139/g74-066

[bib30] Cunningham SJ , Martin RO, Hojem CL, Hockey PA. 2013. Temperatures in excess of critical thresholds threaten nestling growth and survival in a rapidly-warming arid savanna: a study of common fiscals. PLoS One. 8:e74613. 24040296 10.1371/journal.pone.0074613 PMC3767631

[bib31] Darwin C . 1859. The origin of species; and, the descent of man. New Yok (NY): Modern Library.

[bib32] Darwin C . 1868. The variation of animals and plants under domestication. London: John Murray.

[bib33] Dawson RD , Lawrie CC, O'Brien EL. 2005. The importance of microclimate variation in determining size, growth and survival of avian offspring: experimental evidence from a cavity nesting passerine. Oecologia. 144: 499–507. 15891832 10.1007/s00442-005-0075-7

[bib34] Ernst RA , Weathers WW, Smith J. 1984. Effects of heat stress on day-old broiler chicks. Poult Sci. 63:1719–21. 6483736 10.3382/ps.0631719

[bib35] Fan L , Cai T, Xiong Y, Song G, Lei F. 2019. Bergmann’s rule and Allen’s rule in two passerine birds in China. Avian Res. 10:1–11.

[bib36] Freckleton RP , Harvey PH, Pagel M. 2003. Bergmann’s rule and body size in mammals. Am Nat. 161:821–5. 12858287 10.1086/374346

[bib37] Geiser F . 2008. Ontogeny and phylogeny of endothermy and torpor in mammals and birds. Comp Biochem Physiol A: Mol Integr Physiol. 150:176–80. 18499491 10.1016/j.cbpa.2007.02.041

[bib38] Geist V . 1987. Bergmann’s rule is invalid. Can J Zool. 65:1035–8.

[bib39] Geist V . 1990. Bergmann’s rule is invalid: a reply to JD Paterson. Can J Zool. 68:1613–5.

[bib40] Gohli J , Voje KL. 2016. An interspecific assessment of Bergmann’s rule in 22 mammalian families. BMC Evol Biol. 16:1–12. 27760521 10.1186/s12862-016-0778-x PMC5069937

[bib41] Gosler A . 1987. Some aspects of bill morphology in relation to ecology in the great tit Parus major [PhD Dissertation]. Oxford: Oxford University.

[bib42] Gould SJ , Lewontin RC. 1979. The spandrels of San Marco and the Panglossian paradigm: a critique of the adaptationist programme. Proc R Soc Lond B Biol Sci. 205:581–98. 42062 10.1098/rspb.1979.0086

[bib43] Gutiérrez-Pinto N , McCracken KG, Alza L, Tubaro P, Kopuchian C, Astie A, Cadena CD. 2014. The validity of ecogeographical rules is context-dependent: testing for Bergmann’s and Allen’s rules by latitude and elevation in a widespread Andean duck. Biol J Linn Soc Lond. 111:850–62.

[bib44] Hansson L . 1985. Geographic differences in bank voles Clethriononys glareolus in relation to ecogeographical rules and possible demographic and nutritive strategies. Ann Zool Fenn. 22: 319–28.

[bib45] Harrison GA , Morton RJ, Weiner JS. 1959. The growth in weight and tail length of inbred and hybrid mice reared at two different temperatures i. Growth in weight ii. Tail length. Phil Trans R Soc Lond B Biol Sci. 242:479–516.

[bib46] Heath ME . 1983. The effects of rearing-temperature on body composition in young pigs. Comp Biochem Physiol A Comp Physiol. 76:363–6. 6139210 10.1016/0300-9629(83)90338-9

[bib47] Heath ME . 1984. The effects of rearing-temperature on body conformation and organ size in young pigs. Comp Biochem Physiol B. 77:63–72. 6697686 10.1016/0305-0491(84)90224-4

[bib48] Herrington LP , Nelbach JH. 1942. Relation of gland weights to growth and aging processes in rats exposed to certain environmental conditions. Endocrinology. 30:375–86.

[bib49] Huynh TTT . 2005. Heat Stress in Growing Pigs [PhD Dissertation]. Wageningen, the Netherlands: Wageningen University.

[bib50] James FC . 1970. Geographic size variation in birds and its relationship to climate. Ecology. 51:365–90.

[bib51] James FC . 1991. Complementary descriptive and experimental studies of clinal variation in birds. Am Zool. 31:694–706.

[bib52] Johnson JS , Aardsma MA, Duttlinger AW, Kpodo KR. 2018. Early life thermal stress: impact on future thermotolerance, stress response, behavior, and intestinal morphology in piglets exposed to a heat stress challenge during simulated transport. J Anim Sci. 96:1640–53. 29635346 10.1093/jas/sky107 PMC6140855

[bib53] Jonassen TM , Imsland AK, Stefansson SO. 1999. The interaction of temperature and fish size on growth of juvenile halibut. J Fish Biol. 54:556–72.

[bib54] Knudsen B . 1962. Growth and reproduction of house mice at three different temperatures. Oikos. 13:1–14.

[bib55] Krijgsveld KL , Visser GH, Daan S. 2003. Foraging behavior and physiological changes in precocial quail chicks in response to low temperatures. Physiol Behav. 79:311–9. 12834804 10.1016/s0031-9384(03)00117-3

[bib56] Kurnianto E , Shinjo A, Suga D. 1997. Comparison of the three growth curve models for describing the growth patterns in wild and laboratory mice. J Vet Epidemiol. 1:49–55.

[bib57] Lee MM , Chu PC, Chan HC. 1969. Effects of cold on the skeletal growth of albino rats. Am J Anat. 124:239–49. 5774652 10.1002/aja.1001240207

[bib58] Marchini CFP , Silva PL, Nascimento M, Beletti ME, Silva NM, Guimarães EC. 2011. Body weight, intestinal morphometry and cell proliferation of broiler chickens submitted to cyclic heat stress. Int J Poult Sci. 10:455–60.

[bib59] May JD , Lott BD. 2001. Relating weight gain and feed: gain of male and female broilers to rearing temperature. Poult Sci. 80:581–4. 11372706 10.1093/ps/80.5.581

[bib60] Mayr E . 1956. Geographical character gradients and climatic adaptation. Evolution. 10:105–8.

[bib135_1684430283010] McCafferty DJ , Gilbert C, Paterson W, Pomeroy PP, Thompson D, Currie JI, Ancel A. 2011. Estimating metabolic heat loss in birds and mammals by combining infrared thermography with biophysical modelling. Comp Biochem Physiol Part A Mol Integr Physiol. 158:337–45. 10.1016/j.cbpa.2010.09.012 20869456

[bib61] McNab BK . 1971. On the ecological significance of Bergmann’s rule. Ecology. 52:845–54.

[bib62] McNab BK . 2010. Geographic and temporal correlations of mammalian size reconsidered: a resource rule. Oecologia. 164:13–23. 20364270 10.1007/s00442-010-1621-5

[bib63] McQueen A , Klaassen M, Tattersall GJ, Atkinson R, Jessop R, Hassell CJ, Christie M, Victorian Wader Study Group Australasian Wader Studies Group Symonds MR. 2022. Thermal adaptation best explains Bergmann's and Allen's rules across ecologically diverse shorebirds. Nat Commun. 13:4727. 35953489 10.1038/s41467-022-32108-3 PMC9372053

[bib65] Meiri S , Dayan T. 2003. On the validity of Bergmann's rule. J Biogeogr. 30:331–51.

[bib66] Meiri S , Guy D, Dayan T, Simberloff D. 2009. Global change and carnivore body size: data are stasis. Glob Ecol Biogeogr. 18:240–7.

[bib64] Meiri S . 2011. Bergmann's rule–what's in a name?. Glob Ecol Biogeogr. 20:203–7.

[bib67] Mitchell D , Snelling EP, Hetem RS, Maloney SK, Strauss WM, Fuller A. 2018. Revisiting concepts of thermal physiology: predicting responses of mammals to climate change. J Anim Ecol. 87:956–73. 29479693 10.1111/1365-2656.12818

[bib68] Morgan R , Andreassen AH, Åsheim ER, Finnøen MH, Dresler G, Brembu T, Loh A, Miest JJ, Jutfelt F. 2022. Reduced physiological plasticity in a fish adapted to stable temperatures. Proc Natl Acad Sci U S A. 119:2201919119. 10.1073/pnas.2201919119 PMC9295802 35617428

[bib69] Mujahid A , Furuse M. 2009. Oxidative damage in different tissues of neonatal chicks exposed to low environmental temperature. Comp Biochem Physiol A Mol Integr Physiol. 152:604–8. 19256080 10.1016/j.cbpa.2009.01.011

[bib70] Mundim KC , Baraldi S, Machado HG, Vieira FM. 2020. Temperature coefficient (Q10) and its applications in biological systems: beyond the Arrhenius theory. Ecol Modell. 431:109127.

[bib71] NeSmith CC . 1985. The effect of the physical environment on the development of red-winged blackbird nestlings [Msc. Thesis]. Tallahassee: Florida State University.

[bib72] Nord A , Giroud S. 2020. Lifelong effects of thermal challenges during development in birds and mammals. Front Physiol. 11:419. 32523540 10.3389/fphys.2020.00419 PMC7261927

[bib73] Nudds RL , Oswald SA. 2007. An interspecific test of Allen’s rule: evolutionary implications for endothermic species. Evolution. 61:2839–48. 17941837 10.1111/j.1558-5646.2007.00242.x

[bib74] O'Connor RJ . 1975. Growth and metabolism in nestling passerines. In: Peaker M, editor. Avian Physiology, Symposia of the Zoological Society of London. Vol.35. London, UK: Academic Press for the Zoological Society of London. p. 277–306.

[bib76] Ørsted M , Jørgensen LB, Overgaard J. 2022. Finding the right thermal limit: a framework to reconcile ecological, physiological and methodological aspects of CTmax in ectotherm. J Exp Biol. 225:jeb244514. 36189693 10.1242/jeb.244514

[bib77] Ozgul A , Tuljapurkar S, Benton TG, Pemberton JM, Clutton-Brock TH, Coulson T. 2009. The dynamics of phenotypic change and the shrinking sheep of St. Kilda. Science. 325:464–7. 19574350 10.1126/science.1173668 PMC5652310

[bib78] Parsons PA . 2005. Environments and evolution: interactions between stress, resource inadequacy and energetic efficiency. Biol Rev. 80:589–610. 16221331 10.1017/S1464793105006822

[bib79] Pérez JH , Ardia DR, Chad EK, Clotfelter ED. 2008. Experimental heating reveals nest temperature affects nestling condition in tree swallows (*Tachycineta bicolor*). Biol Lett. 4:468–71. 18628112 10.1098/rsbl.2008.0266 PMC2610083

[bib80] Piersma T , Drent J. 2003. Phenotypic flexibility and the evolution of organismal design. Trends Ecol Evol. 18:228–33.

[bib81] Pipoly I , Bókony V, Seress G, Szabó K, Liker A. 2013. Effects of extreme weather on reproductive success in a temperate-breeding songbird. PLoS One. 8:e80033. 24224033 10.1371/journal.pone.0080033 PMC3818280

[bib82] Poole S , Stephenson JD. 1977. Body temperature regulation and thermoneutrality in rats. Q J Exp Physiol Cogn Med Sci. 62:143–9. 585477 10.1113/expphysiol.1977.sp002384

[bib83] Przibram H . 1925. Direkte temperaturabhängigkeit der Schwanzlänge bei Ratten, Mus (Epimys) decumanus Pall. Und M.(E.) rattus L. Die Umwelt des Keimplasmas. XI. Archiv f mikr Anat u Entwicklungsmechanik. 104:434–96.

[bib84] Quinn DE . 1978. The Effect of Developmental Temperature on Morphology, Energy Metabolism, Growth Hormone and Thyroid Stimulating Hormone in Long-Evans Rats [Phd Thesis]. Portland, USA: Portland State University.

[bib85] Rensch B . 1938. Some problems of geographical variation and species-formation. Biol J Linn Soc. 150:275–85.

[bib86] Riek A , Geiser F. 2012. Developmental phenotypic plasticity in a marsupial. J Exp Biol. 215:1552–8. 22496292 10.1242/jeb.069559

[bib87] Riesenfeld A . 1973. The effect of extreme temperatures and starvation on the body proportions of the rat. Am J Phys Anthropol. 39:427–59. 4753139 10.1002/ajpa.1330390311

[bib88] Rodríguez MÁ , Olalla-Tárraga MÁ, Hawkins BA. 2008. Bergmann's rule and the geography of mammal body size in the Western Hemisphere. Glob Ecol Biogeogr. 17:274–83.

[bib89] Rodríguez S , Barba E. 2016. Effects of cool nest microclimates on nestling development: an experimental study with Mediterranean great tits *Parus major*. Ardeola. 63:251–60.

[bib90] Rodriguez S , Barba E. 2016b. Nestling growth is impaired by heat stress: an experimental study in a Mediterranean great tit population. Zool Stud. 55:e40. 31966185 10.6620/ZS.2016.55-40 PMC6511899

[bib91] Romano A , Séchaud R, Roulin A. 2020. Geographical variation in bill size provides evidence for Allen's rule in a cosmopolitan raptor. Global Ecol Biogeogr. 29:65–75.

[bib136_1684430496361] Ronget V, Gaillard JM, Coulson T, Garratt M, Gueyffier F, Lega JC, Lemaître JF. 2018. Causes and consequences of variation in offspring body mass: Meta‐analyses in birds and mammals. Biol Rev. 93:1–27. 28393457 10.1111/brv.12329

[bib92] Ryding S , Klaassen M, Tattersall GJ, Gardner JL, Symonds MR. 2021. Shape-shifting: changing animal morphologies as a response to climatic warming. Trends Ecol Evol. 36:1036–48. 34507845 10.1016/j.tree.2021.07.006

[bib93] Schaeffer PJ , Lindstedt SL. 2013. How animals move: comparative lessons on animal locomotion. Comp Physiol. 3:289–314. 10.1002/cphy.c110059 23720288

[bib94] Scholander PF . 1955. Evolution of climatic adaptation in homeotherms. Evolution. 9:15–26.

[bib95] Sebens KP . 1987. The ecology of indeterminate growth in animals. Annu Rev Ecol Syst. 18:371–407.

[bib99] Serrat MA , King D, Lovejoy CO. 2008. Temperature regulates limb length in homeotherms by directly modulating cartilage growth. Proc Natl Acad Sci U S A. 105:19348–53. 19047632 10.1073/pnas.0803319105 PMC2614764

[bib100] Serrat MA , Schlierf TJ, Efaw ML, Shuler FD, Godby J, Stanko LM, Tamski HL. 2015. Unilateral heat accelerates bone elongation and lengthens extremities of growing mice. J Orthop Res. 33:692–8. 25639189 10.1002/jor.22812 PMC6818498

[bib96] Serrat MA . 2007. Environmentally-determined tissue temperature modulates extremity growth in mammals: a potential comprehensive explanation of Allen's rule [PhD Dissertation]. Kent, USA: Kent State University.

[bib97] Serrat MA . 2013. Allen's rule revisited: temperature influences bone elongation during a critical period of postnatal development. Anat Rec. 296:1534–45. 10.1002/ar.22763 23956063

[bib98] Serrat MA . 2014. Environmental temperature impact on bone and cartilage growth. Comp Physiol. 4:621–55. 10.1002/cphy.c130023 24715562

[bib101] Sheridan JA , Bickford D. 2011. Shrinking body size as an ecological response to climate change. Nat Clim Chang. 1:401–6.

[bib102] Shipley JR , Twining CW, Taff CC, Vitousek MN, Winkler DW. 2022. Selection counteracts developmental plasticity in body-size responses to climate change. Nat Clim Chang. 12: 863–8.

[bib103] Škop V , Guo J, Liu N, Xiao C, Hall KD, Gavrilova O, Reitman ML. 2020. Mouse thermoregulation: introducing the concept of the thermoneutral point. Cell Rep. 31:107501. 32294435 10.1016/j.celrep.2020.03.065 PMC7243168

[bib104] Snedecor JG . 1971. Responses of normal and goitrogen-fed cockerels to different environmental temperatures. Poult Sci. 50:237–43. 5573316 10.3382/ps.0500237

[bib105] Stawski C , Geiser F. 2020. Growing up in a changing climate: how temperature affects the development of morphological, behavioral and physiological traits of a marsupial mammal. Front. Physiol. 11:49. 32116761 10.3389/fphys.2020.00049 PMC7028820

[bib106] Steudel K , Porter WP, Sher D. 1994. The biophysics of Bergmann's rule: a comparison of the effects of pelage and body size variation on metabolic rate. Can J Zool. 72:70–7.

[bib107] Sumner FB . 1909. Some effects of external conditions upon the white mouse. J Exp Zool. 7: 97–155.

[bib109] Sundstroem ES . 1922. Studies on the adaptation of albino mice to an artificially produced tropical climate: I. Effect of the various factors composing a tropical climate on growth and fertility of mice. Am J Physiol. 60:397–415.

[bib108] Sundstroem ES . 1922. Studies on the adaption of albino mice to an artificially produced tropical climate: II. Relations of the body form and especially the surface area to the reactions released by and the resistance to a tropical climate. Am J Physiol. 60:416–24.

[bib110] Suzuki Y , Nijhout HF. 2006. Evolution of a polyphenism by genetic accommodation. Science. 311:650–2. 16456077 10.1126/science.1118888

[bib111] Swain S , Farrell DJ. 1975. Effects of different temperature regimens on body composition and carry-over effects on energy metabolism of growing chickens. Poult Sci. 54:513–20. 1178610 10.3382/ps.0540513

[bib112] Symonds MR , Tattersall GJ. 2010. Geographical variation in bill size across bird species provides evidence for Allen's rule. Am Nat. 176:188–97. 20545560 10.1086/653666

[bib113] Tattersall GJ , Arnaout B, Symonds MR. 2017. The evolution of the avian bill as a thermoregulatory organ. Biol Rev. 92:1630–56. 27714923 10.1111/brv.12299

[bib114] Teplitsky C , Mills JA, Alho JS, Yarrall JW, Merilä J. 2008. Bergmann's rule and climate change revisited: disentangling environmental and genetic responses in a wild bird population. Proc Natl Acad Sci U S A. 105:13492–6. 18757740 10.1073/pnas.0800999105 PMC2533217

[bib115] Teulier L , Rouanet JL, Rey B, Roussel D. 2014. Ontogeny of non-shivering thermogenesis in Muscovy ducklings (*Cairina moschata*). Comp Biochem Physiol A Mol Integr Physiol. 175:82–9. 24862961 10.1016/j.cbpa.2014.05.012

[bib116] Thorington RW Jr . 1970. Lability of tail length of the white-footed mouse, *Peromyscus leucopus noveboracensis*. J Mammal. 51:52–9. 5455373

[bib117] Ton R , Stier A, Cooper CE, Griffith SC. 2021. Effects of heat waves during post-natal development on mitochondrial and whole body physiology: an experimental study in zebra finches. Front Physiol. 554:1–11. 10.3389/fphys.2021.661670 PMC8110927 33986695

[bib118] Verberk WC , Atkinson D, Hoefnagel KN, Hirst AG, Horne CR, Siepel H. 2021. Shrinking body sizes in response to warming: explanations for the temperature–size rule with special emphasis on the role of oxygen. Biol Rev. 96:247–68. 32959989 10.1111/brv.12653 PMC7821163

[bib119] Villarreal JA , Schlegel WM, Prange HD. 2007. Thermal environment affects morphological and behavioral development of *Rattus norvegicus*. Physiol Behav. 91:26–35. 17341426 10.1016/j.physbeh.2007.01.013

[bib120] Wallace AR . 1855. XVIII—on the law which has regulated the introduction of new species. Ann Mag Nat Hist. 16:184–96.

[bib121] Walters RJ , Hassall M. 2006. The temperature-size rule in ectotherms: may a general explanation exist after all? Am Nat. 167:510–23. 16670994 10.1086/501029

[bib122] Watt C , Mitchell S, Salewski V. 2010. Bergmann's rule; a concept cluster? Oikos. 119:89–100.

[bib123] Weaver ME , Ingram DL. 1969. Morphological changes in swine associated with environmental temperature. Ecol. 50:710–3.

[bib125] Weeks BC , Harvey C, Tobias JA, Sheard C, Zhou Z, Fouhey DF. 2023. Bird wings are shaped by thermoregulatory demand for heat dissipation. Biorxiv Preprint. 10.1101/2023.02.06.527306.

[bib126] Weeks BC , Klemz M, Wada H, Darling R, Dias T, O'Brien BK, Probst CM, Zhang M, Zimova M. 2022. Temperature, size and developmental plasticity in birds. Biol Lett. 18:20220357. 36475424 10.1098/rsbl.2022.0357 PMC9727665

[bib127] Wells JC . 2014. Adaptive variability in the duration of critical windows of plasticity: implications for the programming of obesity. Evol Med Public Health. 2014:109–21. 25095791 10.1093/emph/eou019 PMC4148720

[bib128] West-Eberhard MJ . 1989. Phenotypic plasticity and the origins of diversity. Annu Rev Ecol Syst. 20:249–78.

[bib129] Whittow GC , Tazawa H. 1991. The early development of thermoregulation in birds. Physiol Zool. 64:1371–90.

[bib130] Williams JB . 1988. Field metabolism of tree swallows during the breeding season. Auk. 105:706–14.

[bib131] Winther RG . 2000. Darwin on variation and heredity. J Hist Biol. 33: 425–55.

[bib132] Yom-Tov Y , Geffen E. 2011. Recent spatial and temporal changes in body size of terrestrial vertebrates: probable causes and pitfalls. Biol Rev. 86:531–41. 21070587 10.1111/j.1469-185X.2010.00168.x

[bib133] Youngflesh C , Saracco JF, Siegel RB, Tingley MW. 2022. Abiotic conditions shape spatial and temporal morphological variation in North American birds. Nat Ecol Evol. 6:1860–70. 36302998 10.1038/s41559-022-01893-x

